# Hyper-reflective foci changes in RRMS under natalizumab therapy

**DOI:** 10.3389/fimmu.2024.1421755

**Published:** 2024-07-15

**Authors:** Marco Puthenparampil, Elisa Basili, Marta Ponzano, Valentina Annamaria Mauceri, Alessandro Miscioscia, Elisabetta Pilotto, Paola Perini, Francesca Rinaldi, Francesca Bovis, Paolo Gallo

**Affiliations:** ^1^ Department of Neurosciences, University of Padua, Padua, Italy; ^2^ Multiple Sclerosis Centre, Azienda Ospedaliera di Padova, Padua, Italy; ^3^ Department of Health Sciences, Section of Biostatistics, University of Genova, Genova, Italy; ^4^ Ophthalmology Clinic, Azienda Ospedaliera di Padova, Padua, Italy

**Keywords:** multiple sclerosis, natalizumab, optical coherence tomography, retinal hyper-reflective foci, retina

## Abstract

**Introduction:**

Microglia (MG) is suggested to play an immunopathological role of in Multiple Sclerosis (MS). Since hyper-reflective foci (HRF) might mark MG activation, *in vivo* analysis by Optic Coherence Tomography (OCT) in MS patients under disease modifying therapies may help to clarify MS immunopathology as well as drug’s mechanism of intrathecal action.

**Objective:**

To analyze HRF in patients treated with Natalizumab (NTZ), a high efficacy therapy for MS.

**Materials and methods:**

The effect of NTZ on the retina of 36 Relapsing-Remitting MS patients was investigated in a prospective, single-center study. OCT was performed immediately before the first infusion and then between infusion 3 and 4, infusion 6 and 7, infusion 11 and 13. Peripapillary and macular scans were acquired, evaluating peripapillary RNFL thickness, macular volumes (vertical scans), and HRF count (horizontal scan) in Ganglion Cell Layer (GCL), Inner Plexiform Layer (IPL) and Inner Nuclear Layer (INL). Clinical examination was performed every six months.

**Results:**

HRF count significantly increased under NTZ therapy (p<0.001) in both GCL (18.85 ± 6.93 at baseline, 28.24 ± 9.55 after 12 months) and IPL (25.73 ± 7.03 at baseline, 33.21 ± 8.50 after 12 months) but remained stable in INL (33.65 ± 7.76 at baseline, 36.06 ± 6.86 after 12 months, p=0.87), while no relevant modification of pRNFL and macular volumes were observed during the study. EDSS remained stable and no clinical relapse was observed between month 6 and 12.

**Conclusion:**

In RRMS NTZ affects HRF count in GCL and IPL, but not in INL, suggesting that NTZ does not impact on some aspects of MS immunopathology.

## Introduction

Multiple Sclerosis (MS) is a chronic, inflammatory and neurodegenerative disorder, affecting the central nervous system (CNS) (1). Several lines of evidence indicate an autoimmune origin of MS, however, while the role of adaptive immunity in inducing acute CNS inflammation is largely known, the role of tissue-resident macrophage (microglia, MG) is still debated. MG are professional antigen-presenting cells, as well as a source of pro-inflammatory cytokines (2, 3), and an active role of these cells in sustaining smoldering disease (chronic active demyelinating lesions has been suggested (4, 5). Therefore, MG can play pivotal detrimental role in both acute and chronic MS-related pathogenetic mechanisms.

Optical Coherence Tomography (OCT) allows to study *in vivo* retinal microglia activation (6). Indeed, it has been recently demonstrated in an animal model of diabetes that focal Blood-Retina-Barrier (BRB) damage strongly associate with retinal microglia proliferation, and the histologically defined pattern corresponded to the hyper-reflective foci (HRF) observed by OCT (6). Thus, HRF has been proposed as *in-vivo* biomarker of microglia proliferation.

In MS, HRF are increased in patients with active disease (7). Moreover, cortical demyelination load strongly correlates with deep venous complex HRF count (previously counted as HRF INL) (8). Finally, further evidence of microglial origin derived from the analysis of CSF, where monocyte-derived cytokines (CXCL-13 especially) correlated with INL-HRF count (9). To what extent the increase in HRF count may reflect microglia activation induced by a local signal or is the effect of pro-inflammatory mediators (both cells and soluble molecules) crossing the BRB remains to be investigated.

To evaluate the relation between peripheral adaptive immunity cells and HRF, we tested if HRF count was influenced by peripheral immunity (outside-inside hypothesis) or was determined by local microglia activation (outside-inside hypothesis) by evaluating HRF count in MS patients treated with natalizumab (NTZ), a highly effective treatment for MS (10), which strongly reduces the lymphocytes diapedesis into CNS and impacts on BBB damage (11).

## Materials and methods

### Study population

A single-center, prospective, observational, cohort study was conducted in the Multiple Sclerosis Centre of the University Hospital of Padua between January 2022 and April 2023, recruiting patients with a diagnosis of Relapsing-Remitting MS and the indication to start Natalizumab (300 mg of Natalizumab every 4 weeks). OCT was acquired at first therapy infusion (baseline, T0) and at specific timepoint of therapy infusion (between infusion 3 and 4, T1; between infusion 6 and 7, T2; and between infusion 11 and 13, T3) (12). Inclusion criteria were the following: (1) diagnosis of RRMS according to the revised 2017 McDonald criteria with or without positive history of optic neuritis; (2) Natalizumab administration in line with current indication from AIFA (https://www.aifa.gov.it/documents/20142/241044/rcp_tysabri220508.pdf).

Exclusion criteria were the following: (1) any systemic or ophthalmologic disorders affecting the retina other than MS; (2) diagnosis of progressive MS; (3) high-dose steroid therapy in the 28 days preceding first OCT acquisition. Indeed, before starting NTZ and in presence of clinical or radiological (detection of gadolinium enhancing lesion) relapse, patients could had been treated with high dose steroids (Methylprednisolone 1g for 3-5 days).

The study was conducted in agreement with the Declaration of Helsinki and approved by the local Ethic Committee (Comitato Etico per la Sperimentazione Clinica dell’Azienda Ospedaliera di Padova, prot.n. 17760, 17/03/21).

### Spectral-Domain OCT

At all timepoints MS patients underwent spectral-domain OCT (Spectralis; Heidelberg Engineering, Carlsbald, CA; Heidelberg Eye Explorer version 1.7.0.0) examination of both eyes in a dark room without the injection of any mydriatic agent. Two macular scans (vertical for volume evaluation, horizontal for HRF count) 20° x 20° and one peripapillary 3.4 mm ring scan were acquired. The peripapillary 3.4 mm ring scan, centered on the optic nerve head, was used to measure mean global peripapillary RNFL and mean sectorial peripapillary RNFL (pRNFL) thickness (temporal pRNFL, pRNFL-T; supero-temporal RNFL, pRNFL-TS; supero-nasal RNFL, pRNFL-NS; nasal RNFL, pRNFL-N; infero-nasal RNFL, pRNFL-NI; infero-temporal RNFL,pRNFL-TI). Moreover, mean thickness of the papillomacular bundle (pRNFL-PMB) and the relationship between the thickness of the nasal sectors and that one of the temporal sectors were measured. Scans with ART between 90 and 100 frames were considered valid. The 2 macular map scans, automatically centered on the fovea, were composed of 25 linear scans in the High-Resolution Mode. Each scan was performed with the ART (Automatic Real-Time) system to increase image quality and it was settled at 49 frames averaged per B-scan. The retinal layering was obtained using the automatic layering of the Spectralis SD-OCT. The widespread ETDRS (early treatment diabetic retinopathy study) map was used to analyze the different OCT macular parameters: macular area was split in 9 parts, according to the incorporated Spectralis software, by a grid centered on the fovea consisting of a central circular zone with a 1-mm diameter and inner and outer rings of 3 and 6-mm diameter, respectively. The internal and external rings were subdivided into 4 quadrants (superior, inferior, temporal, and nasal). Automatic segmentation software (segmentation technology; Heidelberg Engineering, version 6.3.1.0) was used to identify and measure the volume (for each layer) and thickness (of each quadrant). After the automatic segmentation process, all scans and all layers were carefully revised for algorithm failure. The following retinal slabs, automatically obtained by the incorporated algorithm, were measured in the vertical macular map: macular RNFL (mRNFL); macular ganglion cell layer (mGCL); macular inner plexiform layer (mIPL); macular inner nuclear layer (mINL); macular outer plexiform layer (mOPL); macular outer nuclear layer (mONL). The mGCL and mIPL were then unified in a unique layer (mGCIP). The longitudinal study was conducted by using the automatic rescan mode which guarantee automatic centring of the image basing on the reference scan acquired for each patient. This methodology allows to minimize any errors due to different patient position or movements and different operators. All examinations were checked for sufficient quality using the OSCAR-IB criteria (13). The results are reported in line with the Advised Protocol for OCT Study Terminology and Elements (14, 15).

### Intraretinal Hyperreflective Foci

**Figure 1:**
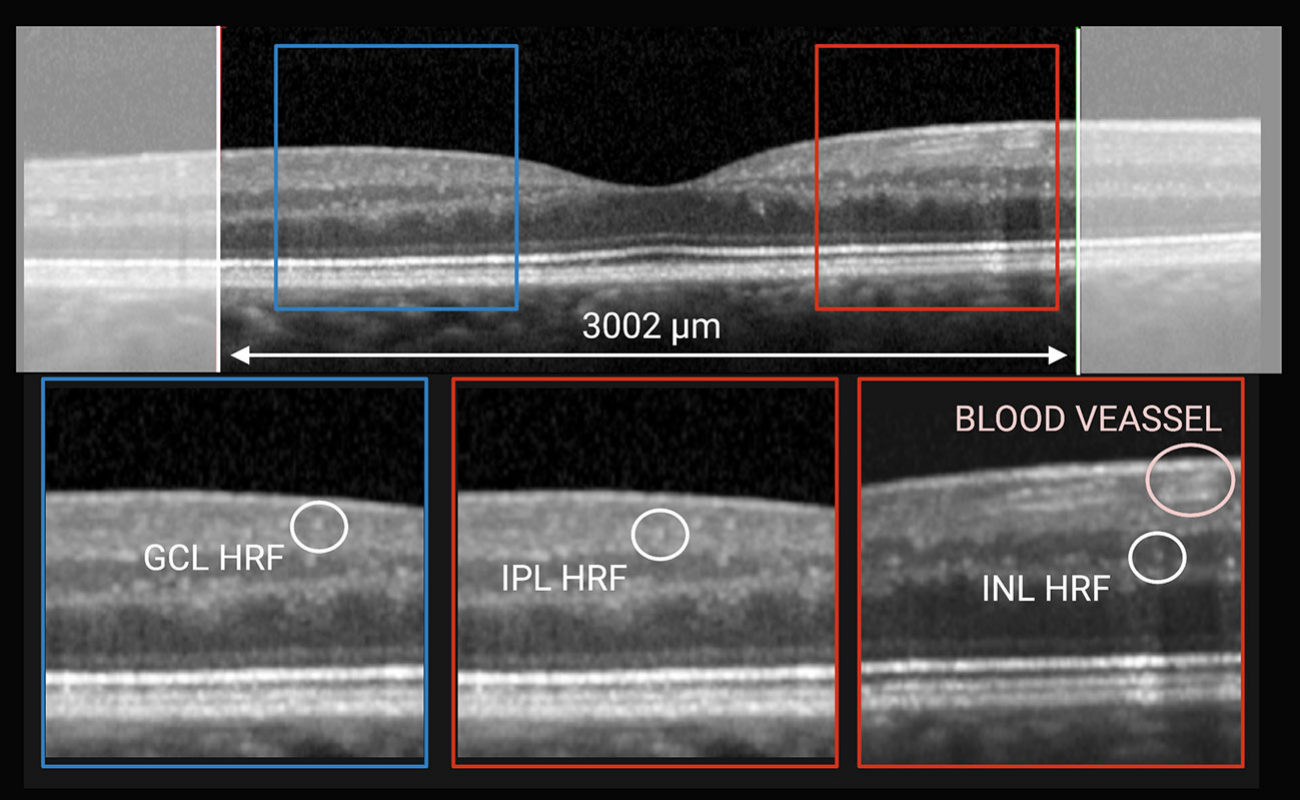
HRF count was performed classifying them according to location. On horizontal macular scan (top) HRF were counted in the area included between 2 perpendicular lines to Bruch membrane traced at 1,500 μm both temporally and nasally from the center of the fovea. Based on their location they were divided into GCL-HRF (bottom left), IPL-HRF (bottom center), and INL-HRF (bottom right). Created with BioRender.com.

According to recent publications (8, 9), the central linear scan of the horizontal macular map, crossing the fovea, was considered for HRF counting. HRF, defined as isolated, small-size (<30 μm), punctiform elements with moderate reflectivity (similar to that of the nerve fiber layer) but without any back shadowing, were counted in the area included between 2 perpendicular lines to Bruch membrane traced at 1,500 μm both temporally and nasally from the center of the fovea. The count was performed in the GCL, IPL and INL separately and results were reported also as GCIPL HRF count (i.e., GCL HRF count + IPL HRF count), as indicated in **Figure 1**. HRF count was determine by consensus by 2 raters, who were blinded to both timepoint and patient’s clinical and radiological outcomes (MP and EB).

### Clinical follow-up

All MS patients were evaluated by trained neurologists (FR and PP), who indicated the EDSS score, at baseline, T1, T2, and T3.

### Statistical analysis

Results are reported as absolute frequency (%) for categorical variables and as mean ± standard deviation for the continuous ones. For all the parameters we performed two mixed effects models with random intercept for subjects and eyes within subjects: one to compare each timepoint to the baseline and one to evaluate the linear relationship with time, expressing the presence of a progressive modification of the parameter. All the models were adjusted for age, sex and use of previous treatments (yes/no). All statistical analyses were performed with the statistical software Stata (v.18; StataCorp); p-values < 0.05 were considered statistically significant.

## Results

### Study population at baseline

Thirty-six RRMS patients (24 female and 12 male) were recruited in the study. The great majority were treatment naïve (29 patients, 80.6%). Indeed, mean age at first OCT was 30.92 ± 12.42 years, while median disease duration was of 1 month (**Table 1**). The main clinical and demographic characteristics of are summarized in **Table 1**. While all patients completed the six-month (T2) follow up, 20 patients (55.6%) had an available OCT scan at T3.

**Table 1:** Study population: demography and clinical parameters.

	RRMS
Sex ratio (F/M)	2.0 (24/12)
Age (years), mean ± sd	30.92 ± 12.42
Disease Duration (months), median (range)	1 (0-375)
EDSS at T0, median (range)	1.0 (0.0-5.5)
EDSS at T1, median (range)	1.0 (0.0-5.5)
EDSS at T2, median (range)	1.0 (0.0-5.0)
EDSS at T3, median (range)	1.0 (0.0-5.0)
**Number of previous treatments**, median (range)	0 (0-3)
*naive patients, N(%)*	29(80.6%)
*1 previous treatment, N(%)*	2(5.5%)
*2 previous treatments, N(%)*	4(11.1%)
*3 previous treatments, N(%)*	1(2.8%)
Last Treatment	
*Interferon β-1a*	1
*DiMethylFumarate*	4
*Fingolimod*	2

EDSS, Expanded Disability status scale.

EDSS at baseline was 1.0 and remained stable during the timepoints (T0 vs T3p=0.10) (**Table 1**). Between baseline and T3 no clinical activity was observed. Three patients had radiological activity at T2: in absence of neutralizing antibodies, they all continue NTZ.

### OCT parameters

Raw peripapillary and macular data are reported in **Supplementary Table 1**. During the follow-up, mINL total volume showed a mild transitory reduction at T1 (β: -0.01, p=0.03) (**Table 2**), which was not confirmed over time (β: -0.01, p= 0.18). However, a trend for temporal inner ring reduction was observed (β: -0.21, p= 0.10) (**Supplementary Table 2**).

**Table 2:** Peripapillary thickness and macular volume values during NTZ treatments.

	T1^A^			T2^A^			T3^A^			Overall^B^		
	β-value	95%CI	p-value	β-value	95%CI	p-value	β-value	95%CI	p-value	β-value	95%CI	p-value
pRNFL-G (μm)	0.18	-0.35-0.70	0.512	0.41	-0.12-0.93	0.128	0.53	-0.13-1.19	0.112	0.19	-0.01-0.38	0.059
pRNFL-PMB (μm)	0.32	-0.38-1.02	0.370	0.23	-0.46-0.93	0.513	-0.39	-1.26-0.49	0.383	-0.06	-0.32-0.20	0.635
pRNFL-NS (μm)	-0.32	-1.62-0.97	0.625	0.36	-0.94-1.66	0.589	0.09	-1.53-1.72	0.910	0.12	-0.37-0.60	0.638
pRNFL-N (μm)	0.12	-0.93-1.16	0.825	0.43	-0.61-1.48	0.416	1.20	-0.11-2.51	0.072	0.35	-0.04-0.74	0.080
pRNFL-NI (μm)	0.27	-1.59-2.14	0.774	1.36	-0.51-3.22	0.154	**3.83**	**1.49-6.17**	**0.001**	**1.11**	**0.41-1.81**	**0.002**
pRNFL-TI (μm)	0.77	-0.83-2.37	0.346	0.90	-0.70-2.50	0.269	-0.08	-2.08-1.92	0.937	0.11	-0.49-0.70	0.724
pRNFL-T (μm)	0.22	-0.53-0.96	0.567	-0.11	-0.85-0.64	0.780	-0.91	-1.85-0.02	0.055	-0.24	-0.52-0.04	0.090
pRNFL-TS (μm)	0.52	-0.72-1.77	0.411	0.49	-0.75-1.74	0.436	0.12	-1.44-1.68	0.882	0.09	-0.38-0.55	0.710
mRNFL (mm^3^)	0.002	-0.01-0.01	0.649	**0.009**	**0.0002-0.02**	**0.044**	0.001	-0.01-0.01	0.844	0.002	-0.001-0.005	0.275
mGCL (mm^3^)	0.004	-0.001-0.01	0.120	0.004	-0.001-0.01	0.161	-0.006	-0.01-0.0004	0.065	-0.001	-0.003-0.001	0.327
mIPL (mm^3^)	0.001	-0.002-0.005	0.553	-0.003	-0.01-0.0004	0.079	-0.002	-0.01-0.002	0.269	-0.001	-0.003-0.0001	0.062
mGCIPL (mm^3^)	0.01	-0.001-0.01	0.082	0.001	-0.01-0.01	0.866	**-0.01**	**-0.02–0.001**	**0.020**	**-0.002**	**-0.004–0.0001**	**0.045**
mINL (mm^3^)	**-0.01**	**-0.01–0.001**	**0.030**	-0.003	-0.01-0.002	0.236	-0.005	-0.01-0.001	0.125	-0.001	-0.003-0.001	0.178
mOPL (mm^3^)	-0.001	-0.01-0.01	0.842	-0.004	-0.01-0.01	0.485	0.003	-0.01-0.02	0.619	0.0001	-0.004-0.004	0.948
mONL (mm^3^)	0.01	-0.002-0.02	0.086	0.01	-0.004-0.02	0.155	-0.01	-0.02-0.01	0.330	-0.001	-0.01-0.004	0.724
mOPNL (mm^3^)	0.01	0.002-0.02	0.011	0.01	-0.003-0.01	0.174	-0.005	-0.02-0.01	0.343	-0.001	-0.004-0.002	0.604

Results are shown as beta(95% CI) and p-values from the mixed effects models with random intercept to compare each timepoint to the baseline (A) and to evaluate the overall change over time (B).

pRNFL, peripapillary RNFL; G, globa; T, temporal; I, Inferior; N, nasal; S, Superior; mRNFL, macular RNFL; mGCL, macular ganglion cell layer; mIPL, macular inner plexiform layer; mGCIP, mGCL + mIPL; mINL, macular inner nuclear layer; mOPL, macular outer plexiform layer; mONL, macular outer nuclear layer. T1, between infusion 3 and 4; T2, between infusion 6 and 7; T3, between infusion 11 and 13. Significant values are in bold.

On the other hand, mGCIP total volume reduced at T3 (β: -0.01, p=0.02) (**Table 2**). In addition, mGCIPL total volume showed a significantly linear reduction over time (β: -0.01, p= 0.05) (**Table 2**).

**Figure 2:**
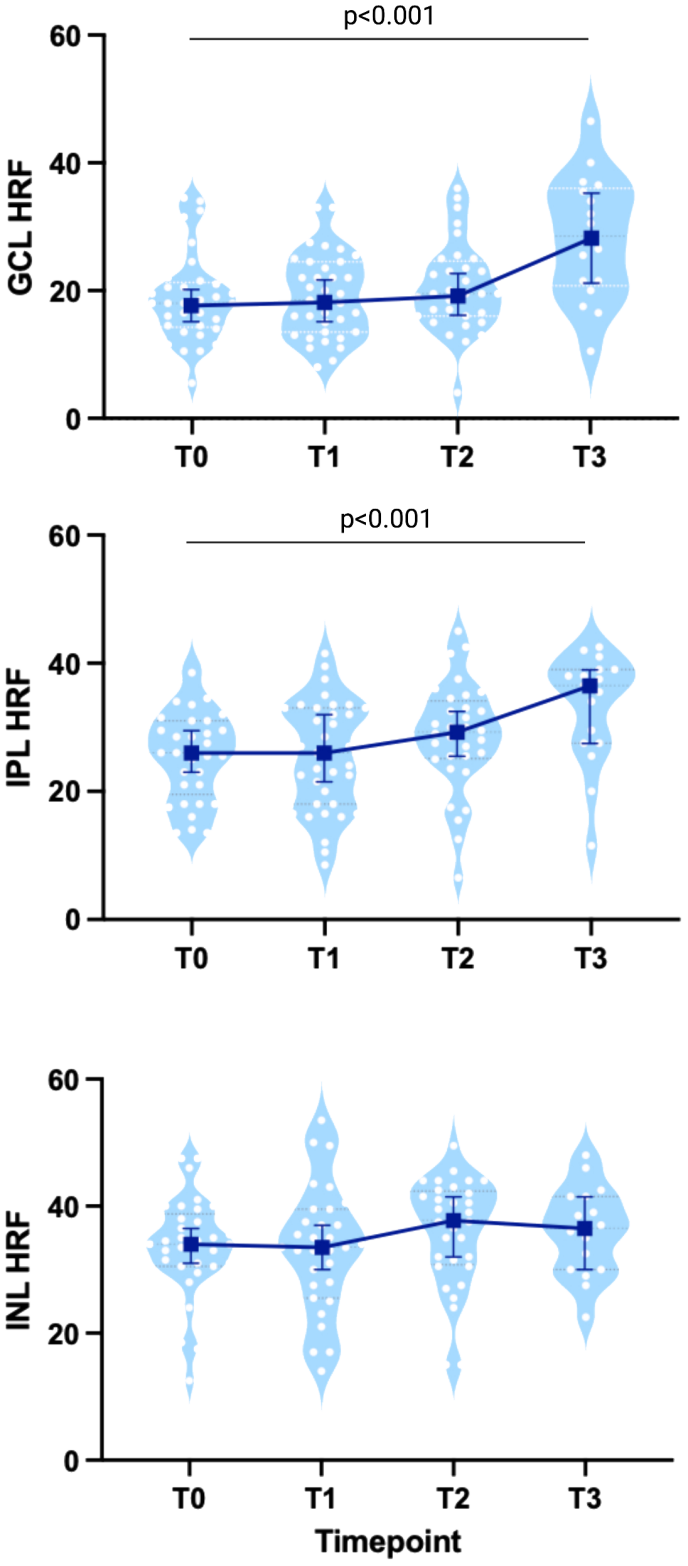
HRF count in MS patients treated with Natalizumab. Both GLC- and IPL-HRF count progressively increased, especially at T3, while INL-HRF count was stable. Each white dot represents the mean value between the 2 eyes from each patient at each timepoint, shown on a light blue violin-plot. Blue bars represent the median values with IC95%. Created with BioRender.com.

HRF count showed a significant progressive increase in both GCL (19.75 ± 7.68 at baseline; 20.81 ± 6.4 at T1, p=0.53; 21.18 ± 7.63 at T2, p=0.39; 29.09 ± 9.64 at T3, p<0.001) and IPL (26.95 ± 8.14 at baseline, 27.89 ± 8.67 at T1, p=0.75; 29.5 ± 9.18 at T2, p=0.06; 34.21 ± 7.63 at T3, p<0.001) (**Figure 2**, **Table 3**; **Supplementary Table 1**), which was further when the overall trend was assessed (β: 1.91 and β: 1.67 respectively, both p<0.001). Nevertheless, HRF INL count a showed a mild but not significant increase (β: 0.07, p=0.87).

**Table 3:** HRF count during NTZ treatment.

	T1 ^A^			T2 ^A^			T3 ^A^			Overall ^B^		
	β-value	95%CI	p-value^a^	β-value	95%CI	p-value^a^	β-value	95%CI	p-value^a^	β-value	95%CI	p-value^b^
HRF GCL	0.69	-1.47-2.86	0.529	0.96	-1.24-3.15	0.393	**7.78**	**5.06-10.49**	**<0.001**	**1.91**	**1.07-2.74**	**<0.001**
HRF IPL	0.34	-1.78-2.46	0.754	2.04	-0.11-4.20	0.063	**5.67**	**3.01-8.33**	**<0.001**	**1.67**	**0.87-2.47**	**<0.001**
HRF GCIP	1.02	-2.67-4.70	0.588	2.95	-0.79-6.70	0.122	**13.29**	**8.67-17.91**	**<0.001**	**3.53**	**2.11-4.94**	**<0.001**
HRF INL	1.39	-0.65-3.43	0.182	1.49	-0.58-3.57	0.158	-0.58	-3.14-1.98	0.658	0.07	-0.71-0.84	0.868

Results are shown as beta (95% CI) and p-values from the mixed effects models with random intercept to compare each timepoint to the baseline (A) and to evaluate the overall change over time (B).

HRF, Hyper-reflective Foci; GCL, Ganglion-Cell Layer; IPL, Inner Plexiform Layer; INL, Inner Nuclear Layer. T1, between infusion 3 and 4; T2, between infusion 6 and 7; T3, between infusion 11 and 13. Significant values are in bold.

## Discussion

Several lines of evidence suggest a pivotal role of MG in MS immunopathology (16). In an inside-outside view of MS immune-pathology, local MG activation drives endothelial cells changes and blood-brain barrier (BBB) damage, thus favoring autoreactive lymphocyte recruitment in the CNS (3). In an outside-inside model, autoreactive peripheral cells and pro-inflammatory soluble mediators act on BBB, leading to its activation and breakdown, facilitating autoreactive lymphocyte recruitment and inducing pro-inflammatory changes in MG that becomes the side-partner (production pro-inflammatory cytokines and effective antigen presentation) of adaptive immunity (16). To test the effect of peripheral adaptive immunity on HRF count (the outside-inside hypothesis) we evaluated the retinal modification occurring in MS patients treated with natalizumab, surprisingly observing a significant increase of HRF count in the inner retina of NTZ-treated MS patients in absence of clinical or radiological disease activity.

Indeed, natalizumab strongly reduces the lymphocyte recruitment in the CNS, as demonstrated by several studies (11, 17). Activated MG is found at the edge of chronic active lesions and though to play a role in sustaining smoldering disease and neurodegeneration (4). The evaluation of microglia activity *in vivo* is limited by the expansive and complex methodology (i.e., PET-MRI) needed (18, 19). Recently, OCT was demonstrated to be able to identify HRF, nodules of activated microglia, in the inner retina, (6). In previous studies, we investigated the association of HRF with inflammatory parameters and found a strong correlation with cortical demyelination (8). Indeed, similarly with retinal pathology, also cortical demyelination has spare cells within its contest (20, 21).

We observed a sustained increased HRF count in GCL and IPL, a phenomenon especially evident after 6 months of treatment, while INL HRF count did not change. This unexpected finding seems to indicate that the mechanisms of retinal MG activation and proliferation are not switched-off by the effect of NTZ on lymphocytes and, thus, are probably unlinked to peripheral adaptive-immunity.

The absence of any significant effect of NTZ on INL HRF count, which strongly associated with cortical inflammation (8), is in line with the observation obtained by PET in a small number of NTZ-treated MS patients of a decreased MG activation in white matter lesions and normal appearing white matter, but not in cortical grey matter (19). It must be considered that the great majority of INL HRF are located at the border of this layer, as are the deep vascular complex (22), further suggesting a strong link between the BRB disfunction and increased INL HRF count in MS. Intriguingly, while the effect of NTZ on CSF-infiltrating cells is largely demonstrated (11, 17), its action on BBB is more questioned. Indeed, after one year the 50% of patients with pathological albumin quotient (Q_ALB_) had persisting pathological values (11). In a different study, 2 patients had persisting pathological Q_ALB_ despite NTZ therapy (23). These observations suggest that the effect of NTZ on BBB damage (identified by pathological Q_ALB_) is heterogeneous or, at least, requires time (more than 48 months). Likely, also BRB disfunction is not significantly impacted by NTZ, thus linking the persistent increased INL HRF count to a sustained BRB damage. A persistent BRB dysfunction might also explain the microcystic macular oedema observed in the INL in later disease phases of MS, and in patients with higher Multiple Sclerosis Severity Scores, a measure of disease progression (24, 25). Since a percentage of MS patients treated for long time with NTZ present a disability progression independent of relapse (clinical and/or radiological) activity (PIRA) (5), the follow-up of our patients will clarify if HRF increase is linked to a higher risk of disease progression. In the present study we were not able to clearly exclude the effect of peripheral cytokines in inducing modification in the BRB. However, the contribute of soluble mediators in serum seems to be not relevant in MS, especially when compared to systemic autoimmune disorder, such as Systemic Lupus Erythematosus, since serum cytokines are not clearly increased in MS (26).

Moving on to INL macular volume, the absence of any significant effect of natalizumab (even if a mild trend was observed in temporal inner ring, which calls for further studies with including more patients) is in line with the hypothesis of a chronic BRB dysfunction, but apparently not with a recent study (12). However, the absence of effect on both mINL volume and HRF count is in line with a previously observed correlation between these two parameters (8) and with data on the effect of fingolimod, a molecule that crosses the BBB and BRB barrier, acting on microglia (12). In addition, no effect of NTZ treatment was found on peripapillary RNFL and total macular volume (27).

The NTZ-associated increased in HRF count in IPL and GCL is intriguing and worth of further investigation. Indeed, we previously showed that GCL HRF were not increased in MS (8). Since superficial vascular complex modifications have been also described in MS (28), whether this activation is linked to a progressive disfunction of superficial vascular complex BRB or represent an anti-inflammatory polarization of local microglia needs to be clarified. Since GCL loss was associated to a more pronounced humoral response in the CSF (29) and that superficial vascular complex have been linked with activated B-cells infiltration in CSF (28), it could be hypothesized that NTZ, while reducing CSF (and CNS) lymphocyte infiltration (17), do not affect vascular and cellular changes in GCL. In this case HRF increase in GCL and IPL might have a protective significance.

Our study presents a few limitations, that have been partially discussed. First, our work is limited by the cohort of NTZ-treated MS patients recruited, which did not allow to identify small retinal modifications, especially on macular volumes, that could occur a cohort of MS patients with very short disease duration. Second, we cannot depict the proinflammatory or anti-inflammatory profile of the retinal MG (20). Moreover, since we did not explore any molecular marker of peripheral immunity, we cannot exclude the influence of soluble factors in serum in BRB dysfunction. Third, we do not include any control cohort. However, in our hypothesis HRF INL might constitute an independent outcome to test the efficacy of drugs on BRB dysfunction/microglia activation, since they are increased in MS, especially in those patients that need to start highly effective treatments. Indeed, the INL HRF count we observed (33.65) was higher than our previous cohorts (19.55 and 17.5) (8, 9). Moreover, we chose NTZ considering the low impact on mechanisms on the other side of the barriers to better understand the origin of HRF. In addition, we could not perform any subgroup analysis (naïve vs not naïve rather than radiologically active vs stable) due to the low number of patients in at least one group. However, the low detection of relapse activity (both clinical and radiological) is completely in line with our previous data on NTZ cohort (30). Finally, it has to been pointed out that our patients were motivated at the beginning to follow the study protocol. However, about 50% did not want to perform the OCT at month 12, when MS was clinically and radiologically stable.

In conclusion, our findings demonstrate that INL HRF count are not decreased by NTZ treatment, confirming that HRF are not induced by peripheral inflammation. In addition, we demonstrate that NTZ, one of the most effective treatments for MS, might not properly target some aspects of MS immunopathogenesis. Therefore, HRF evaluation *in vivo* may help to evaluate the mechanism(s) of MG activation that is thought to drive disease progression in Multiple Sclerosis.

## Data availability statement

The raw data supporting the conclusions of this article will be made available by the authors, without undue reservation.

## Ethics statement

The study was conducted in agreement with the Declaration of Helsinki and approved by the local Ethic Committee (Comitato Etico per la Sperimentazione Clinica dell’Azienda Ospedaliera di Padova, prot.n. 17760, 17/03/21).

## Author contributions

MPu: Conceptualization, Data curation, Funding acquisition, Methodology, Resources, Supervision, Writing – original draft, Writing – review & editing. EB: Data curation, Methodology, Project administration, Resources, Writing – original draft. MPo: Data curation, Formal analysis, Methodology, Writing – original draft. VM: Data curation, Methodology, Resources, Writing – original draft. AM: Conceptualization, Writing – original draft. EP: Conceptualization, Writing – original draft. PP: Conceptualization, Writing – original draft. FR: Conceptualization, Methodology, Writing – original draft. FB: Data curation, Formal analysis, Methodology, Writing – original draft. PG: Conceptualization, Funding acquisition, Supervision, Writing – original draft, Writing – review & editing.
