# Supplementary material for: Hyper-reflective foci changes in RRMS under natalizumab therapy
Source: Front Immunol. 2024 Jul 15;15:1421755. doi: 10.3389/fimmu.2024.1421755 (PMC11284017; doi:10.3389/fimmu.2024.1421755)
Supplement: Supplementary file 1 [file Table_1.pdf]

Supplementary Table 1. Peripapillary and macular values (mean± standard deviation) at each time-point.

	**Baseline (T0)**	**T1**	**T2**	**T3**
pRNFL-G (μm)	95.35 ± 16.16	97.17 ± 13.62	97.00 ± 14.12	89.70 ± 26.50
pRNFL-PMB (μm)	46.57 ± 12.01	47.69 ± 11.13	47.00 ± 10.95	42.63 ± 14.80
pRNFL-NS (μm)	112.29 ± 19.23	113.49 ± 17.22	114.13 ± 17.28	104.43 ± 30.40
pRNFL-N (μm)	71.90 ± 15.05	73.00 ± 13.40	73.47 ± 13.64	69.45 ± 22.41
pRNFL-NI (μm)	116.06 ± 24.83	118.79 ± 24.26	119.24 ± 25.30	110.65 ± 40.33
pRNFL-TI (μm)	132.99 ± 29.29	136.04 ± 28.07	135.04 ± 28.23	124.10 ± 40.66
pRNFL-T (μm)	61.38 ± 15.94	62.63 ± 14.76	61.69 ± 14.77	56.60 ± 19.05
pRNFL-TS (μm)	134.46 ± 26.93	137.80 ± 24.47	137.30 ± 24.82	126.28 ± 39.28
mRNFL (mm^3)	0.80 ± 0.11	0.80 ± 0.12	0.81 ± 0.12	0.81 ± 0.10
mGCL (mm^3)	1.06 ± 0.15	1.07 ± 0.15	1.06 ± 0.15	1.07 ± 0.12
mIPL (mm^3)	0.89 ± 0.11	0.89 ± 0.11	0.88 ± 0.11	0.89 ± 0.08
mGCIPL (mm^3)	1.95 ± 0.26	1.96 ± 0.26	1.95 ± 0.26	1.96 ± 0.20
mINL (mm^3)	0.97 ± 0.10	0.96 ± 0.10	0.97 ± 0.10	0.99 ± 0.05
mOPL (mm^3)	0.81 ± 0.10	0.81 ± 0.09	0.81 ± 0.10	0.83 ± 0.07
mONL (mm^3)	1.73 ± 0.23	1.73 ± 0.23	1.74 ± 0.23	1.76 ± 0.15
mOPNL (mm^3)	2.54 ± 0.28	2.54 ± 0.28	2.55 ± 0.28	2.59 ± 0.14
HRF GCL	18.85 ± 6.93	19.03 ± 6.47	20.52 ± 6.96	28.24 ± 9.55
HRF IPL	25.73 ± 7.03	25.50 ± 8.59	28.58 ± 8.48	33.21 ± 8.50
HRF INL	33.65 ± 7.76	33.43 ± 10.56	35.95 ± 8.40	36.06 ± 6.86
HRF GCIP	44.58 ± 13.21	44.53 ± 14.17	49.09 ± 14.55	61.44 ± 16.74
