# Supplementary material for: Hyper-reflective foci changes in RRMS under natalizumab therapy
Source: Front Immunol. 2024 Jul 15;15:1421755. doi: 10.3389/fimmu.2024.1421755 (PMC11284017; doi:10.3389/fimmu.2024.1421755)
Supplement: Supplementary file 2 [file Table_2.pdf]

Supplementary Table 2. Temporal and nasal macular inner and outer ring volumes. Results are shown as beta(95% CI) and p-values from the mixed effects models with random intercept to evaluate the overall change over time.

**Variable**	**β**	**95%CI**	**p-value**
mGCL Nasal Inner Ring	-0.01	-0.16; 0.14	0.868
mGCL Nasal Outer Ring	-0.08	-0.20; 0.03	0.150
mGCL Temporal Inner Ring	-0.02	-0.20; 0.17	0.864
mGCL Temporal Outer Ring	-0.04	-0.26; 0.17	0.688
mIPL Nasal Inner Ring	-0.14	-0.32; 0.04	0.126
mIPL Nasal Outer Ring	-0.01	-0.10; 0.09	0.901
mIPL Temporal Inner Ring	0.04	-0.12; 0.19	0.633
mIPL Temporal Outer Ring	-0.10	-0.22; 0.03	0.125
mINL Nasal Inner Ring	0.14	-0.18; 0.47	0.395
mINL Nasal Outer Ring	0.05	-0.12; 0.23	0.569
mINL Temporal Inner Ring	-0.21	-0.46; 0.04	0.093
mINL Temporal Outer Ring	-0.002	-0.15; 0.14	0.981
